# Deep learning-based evaluation of the severity of mitral regurgitation in canine myxomatous mitral valve disease patients using digital stethoscope recordings

**DOI:** 10.1186/s12917-025-04802-z

**Published:** 2025-05-08

**Authors:** Soh-Yeon Lee, Sully Lee, Se-Hoon Kim, HyeSun Chang, Won-Yang Cho, Min-Ok Ryu, Jihye Choi, Hwa-Young Yoon, Kyoung-Won Seo

**Affiliations:** 1 Department of Veterinary Clinical Science, Laboratory of Veterinary Internal Medicine, College of Veterinary Medicine, Seoul National University, Seoul, 08826 Republic of Korea; 2 Smartsound Corporation, Seoul, Korea; 3 Department of Veterinary Medical Imaging, College of Veterinary Medicine, Seoul National University, Seoul, 08826 Republic of Korea

**Keywords:** Deep learning, Mitral regurgitation, Canine, Myxomatous mitral valve disease, Digital stethoscope, Phonocardiogram

## Abstract

**Background:**

Myxomatous mitral valve disease (MMVD) represents the most prevalent cardiac disorder in dogs, frequently resulting in mitral regurgitation (MR) and congestive heart failure. Although echocardiography is the gold standard for diagnosis, it is an expensive tool that involves significant clinical training to ensure consistent application. Deep learning models offer an innovative approach to assessing MR using digital stethoscopic recordings, enabling early screening and precise prediction. Thus, in this study, we evaluated the effectiveness of a convolutional neural network 6 (CNN6) in providing an objective alternative to traditional methods for assessing MR. This study, conducted at the Seoul National University Veterinary Medicine Teaching Hospital, included 460 dogs with MMVD, classified according to the American College of Veterinary Internal Medicine guidelines. Phonocardiogram signals were recorded using digital stethoscopes and analyzed using the deep models CNN6, patch-mix audio spectrogram transformer (PaSST), and residual neural network (ResNET38), which were trained to categorize MR severity into mild, moderate, and severe based on MINE score. Performance metrics were calculated to evaluate model effectiveness.

**Results:**

The CNN6-Fbank model achieved an accuracy of 94.12% [95% confidence interval (CI): 94.11–93.12], specificity of 97.30% (95% CI: 97.30–97.34), sensitivity of 94.12% (95% CI: 93.74–94.50), precision of 92.63% (95% CI: 92.29–92.97), and F1 score of 93.32% (95% CI: 93.05–93.59), outperforming the PaSST and ResNet38 models overall and demonstrating robust performance across most metrics.

**Conclusions:**

Deep learning models, particularly CNN6, can effectively assess MR severity in dogs with MMVD using digital stethoscope recordings. This approach provides a rapid, noninvasive, and reliable adjunct to echocardiography, potentially enhancing diagnosis and outcomes. Future studies should focus on broader clinical validation and real-time application of this technology.

**Supplementary Information:**

The online version contains supplementary material available at 10.1186/s12917-025-04802-z.

## Background

Myxomatous mitral valve disease (MMVD) is the most prevalent cardiac condition in dogs, accounting for approximately 75% of heart disease cases in small-to-medium-sized breeds [1], and can lead to mitral regurgitation (MR) and congestive heart failure (CHF). MMVD is characterized by the thickening and elongation of the mitral valve leaflets and chordae tendineae [1]. Its clinical presentations vary widely, ranging from asymptomatic to severe heart failure, with disease progression being notably unpredictable [1–3]. Early detection and accurate assessment of MR are crucial for effective management and treatment and significantly affect the health and quality of life of affected dogs [4, 5].

MR resulting from MMVD is a major contributor to cardiovascular morbidity in dogs, underscoring the need for meticulous monitoring [6]. The progression of MMVD is closely associated with factors such as age, MR severity, and degree of valvular degeneration [7–10]. Notably, findings from human cardiology studies suggest that precise assessments of regurgitant volume and effective orifice area may predict the onset of CHF [11] in dogs, emphasizing the critical role of MR monitoring in the effective management of MMVD.

The American College of Veterinary Internal Medicine (ACVIM) guidelines are commonly used to clinically classify dogs with MMVD [4]. While the ACVIM classification is widely used to stage dogs with MMVD, it does not provide a quantitative assessment of MR severity. To address this, the Mitral INsufficiency Echocardiographic (MINE) score was recently proposed as a simple, objective tool incorporating four echocardiographic parameters—LA/Ao ratio, LVIDDn, fractional shortening (FS), and E-wave peak velocity. The MINE score has been associated with survival outcomes and may complement existing staging systems by offering additional prognostic information^2^. Echocardiographic assessment is essential for understanding the clinical and hemodynamic status, aiding in the prediction of CHF [12, 13] and the evaluation of patient prognosis [14]. Echocardiography remains the gold standard for diagnosing and monitoring MR severity and provides essential information on valve morphology, regurgitation volume, and ventricular function.

In the echocardiographic evaluation of MMVD severity in dogs, assessments include evaluation of cardiac remodeling. This is indicated by definitive criteria, including enlargement of left atrium and ventricle. Conversely, MR quantification is evaluated through several metrics: regurgitation jet size via color Doppler, effective regurgitant orifice area, proximal isovelocity surface area, vena contracta width, and regurgitant fraction [13, 14]. Furthermore, estimation of left ventricular filling pressure is achieved by analyzing mitral inflow patterns, isovolumetric relaxation time, pulmonary venous flow, regurgitant jet profiles, and various tissue Doppler echocardiographic variables [12, 15]. However, many of these methods are time-consuming, require multiple measurements, and are subject to method- and operator-dependent errors (intraobserver and interobserver variability), necessitating the skill of a well-trained operator [2]. Consequently, simpler and less technically demanding methods applicable to veterinary practice would be useful [16].

Auscultation, the practice of detecting mechanical vibrations from the body surface within an audible frequency range, is affected by variability owing to factors such as age-related hearing decline and differences in professional training [17]. Additionally, traditional stethoscopes are limited by human auditory constraints, including a lack of sensitivity to low frequencies, slow reactions to brief sonic events, and masking of softer sounds by louder nearby noises [17]. In human medicine, the diagnostic prominence of auscultation has decreased with the increase in coronary artery disease, reduction in rheumatic valvular disease, and widespread use of advanced cardiac imaging techniques such as Doppler echocardiography [18]. Conversely, in veterinary cardiology, valvular heart diseases such as MMVD are common, highlighting the continued significance of auscultation. Nonetheless, a weak correlation remains between systolic murmur intensity and the severity of regurgitation in dogs [19, 20].

Phonocardiography (PCG) research has shown that MR in dogs leads to changes in heart sounds beyond murmurs [21], with significant findings related to the S3 sound [22–24] and increased intensity of the S1 sound in cases of MR [25, 26]. These findings indicate the potential of artificial intelligence (AI)-assisted digital stethoscopes for detecting sounds inaudible to the human ear. The development of AI-assisted digital auscultation technology offers the promise of overcoming traditional auscultation challenges by providing more accurate assessments of MR severity through the analysis of audible and infrasonic PCG data [27]. Although this technology is not widely used in veterinary practice, it has the potential to significantly improve MR evaluation in dogs, serving as a valuable complement to traditional echocardiography.

Digital stethoscopes represent a significant advancement in enabling the recording and digital analysis of heart sounds. These devices can store heart sounds for later analysis, facilitate the sharing of recordings with specialists for second opinions, and use software to analyze sounds for MR characteristics [28]. In human medicine, digital stethoscopes have demonstrated improved diagnostic outcomes in human medicine, particularly in noisy clinical environments [29]. Similarly, in veterinary practice, digital stethoscopes can improve the accuracy of cardiac assessments by providing objective data and minimizing interobserver variability. Thus, this technology holds significant promise in enhancing human and veterinary cardiology by providing more precise and reliable diagnostic outcomes.

The integration of Artificial Intelligence (AI) and deep learning with digital stethoscopes further enhances their diagnostic potential. AI algorithms, trained on large datasets, are capable of accurately predicting disease outcomes and supporting in clinical decision-making [30, 31]. In the context of MMVD, AI has been employed to analyze echocardiographic data and heart sounds, providing a nuanced understanding of disease progression [32]. For example, AI algorithms have successfully detected and classified heart murmurs in humans, differentiating between benign and pathological murmurs [33]. The application of AI in veterinary medicine is becoming increasingly diverse, encompassing the detection of canine hyperadrenocorticism, classification of various forms of cancer, identification of retinal atrophy, assessment of cardiac enlargement, assistance in radiology, and prediction of seizures in epileptic dogs [34–37]. The diagnostic potential in canine patients can be considerably increased by utilizing these developments in veterinary medicine [28].

In this study, therefore, we aimed to develop an assistive tool using deep learning algorithms to evaluate the severity of MR in dogs diagnosed with MMVD using digital stethoscopic recordings. We hypothesized that these algorithms could reliably interpret heart-sound recordings, offering a noninvasive and accessible method to assist veterinarians in grading MR severity.

## Methods

### Clinical study design

This study was conducted at the Seoul National University Veterinary Medicine Teaching Hospital (SNU VMTH) between May 2022 and August 2023. Eligible dogs were those with a confirmed diagnosis of MMVD, accompanied by comprehensive echocardiographic reports and informed consent obtained from their owners. Prior to participation, the owners received detailed information regarding the objectives of the study and data utilization. All experimental protocols were approved by the Institutional Animal Care and Use Committee of Seoul National University (SNU-220602–1–2). The study was conducted in strict accordance with the Korean Animal Protection Act and the regulatory guidelines of Seoul National University.

**Fig. 1:**
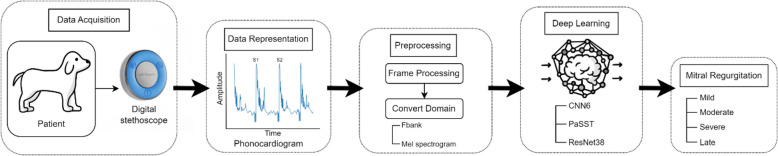
Overview of deep learning-based analysis for mitral regurgitation severity in myxomatous mitral valve disease patients. This figure describes a systematic approach for collecting, preprocessing, and analyzing PCG data from dogs with MMVD to classify MR severity of mitral regurgitation. Data were acquired using a digital stethoscope and preprocessed, visualized, and analyzed using deep learning models. CNN, convolutional neural network; Fbank, filter bank; MMVD, myxomatous mitral valve disease; PaSST, patch-mix audio spectrogram transformer; PCG, phonocardiogram; ResNet, residual neural network

Heart sounds were recorded using the WP-100 digital stethoscope by WITHaPET, which connects to a mobile device via Bluetooth. The recordings were collected through the WITHaPET application, a proprietary software developed by the WP-100 manufacturer for acquiring and storing heart and lung sounds. The WP-100 utilizes a digital MEMS microphone to capture high-quality auscultation sounds, which were then processed into PCGs. Subsequently, the PCGs were employed as the primary input for a deep learning-based classifier to assess the MR severity. Concurrently, the mitral insufficiency echocardiographic (MINE) score [2], an echocardiographic metric that predicts prognosis and survival by correlating higher scores with increased cardiac risk, was used to classify the MR severity into mild, moderate, severe, and late stages(Fig. 1). Further methodological details are presented in the following sections.

The classifier, developed using deep learning models, analyzed PCG signals to classify the MR severity. Features extracted from the PCG signals were employed in classification algorithms to predict the severity of MR with high accuracy. The classifier performance was evaluated using statistical techniques, including the calculation of accuracy, specificity, sensitivity, precision, and F1 score.

## Comprehensive data collection and evaluation methods

### Variables collected

Clinical records from the SNU VMTH were collected from the dogs enrolled in the study. The study participants underwent comprehensive evaluations, including physical examinations, chest radiography, and echocardiographic assessments. The extracted data included breed, sex, age, body condition score, and echocardiographic measurements. While ACVIM staging was used to define the study population, it was not employed as a variable in the analysis or severity classification. The ACVIM classification system has been verified and updated in accordance with the most recent guidelines [4].

### Stethoscope recordings

Heart sounds were recorded by trained veterinary practitioners using a standardized procedure to ensure consistency among all participants. Every dog participated in a 30-s session of PCG recordings while standing. Recordings were acquired from four standard auscultation positions corresponding to the major cardiac valves: aortic, mitral, pulmonary, and tricuspid. Efforts were made to ensure that each patient remained calm and still, showing no indications of rapid breathing or trembling, to optimize the efficiency of sound capture. Auscultatory recordings were reviewed by the study investigators for quality control. The study personnel performing algorithm development were unaware of the detailed echocardiographic information. The specific auscultation sites were as follows.

- Mitral valve: 5 th intercostal space at the costochondral junction of the left apex.
- Aortic valve: precisely above the costochondral junction in the 4 th intercostal space.
- Pulmonary valve: between the 2nd and 4 th intercostal space positioned just above the sternum.
- Tricuspid valve: between the 3rd and 5 th intercostal space near the costochondral junction.

Before each recording, the stethoscope was carefully positioned at each site to confirm the precise location of the valves. Cardiac sounds were methodically recorded from left to right at the correct locations.

### Echocardiographic data

Echocardiographic examinations were performed on the canines by clinicians at the Department of Radiology at SNU VMTH. These examinations used phased-array transducers with a frequency range of 2–9 MHz and single-lead electrocardiography simultaneously. Imaging was performed from right and left parasternal views using two-dimensional, M-mode, and Doppler imaging techniques [38].

The MINE scoring system, which integrates four key echocardiographic metrics, was used to assess the severity of MR: (a) left atrium to aorta ratio, obtained from the right parasternal short-axis view [39]; (b) left ventricular end-diastolic diameter normalized for body weight, obtained in M-mode from the same perspective [39]; (c) left ventricular fractional shortening, measured in a similar manner [40]; and (d) peak E-wave transmitral flow velocity, measured by pulsed-wave Doppler from the left apical four-chamber view [12].

### Digital stethoscope specification

A WP-100 digital stethoscope was used for auscultation. This device features Bluetooth BLE 5.0, which facilitates seamless wireless data acquisition. Equipped with a USB C-type connector, the WP-100 utilizes MEMS microphones as its primary sensors. The stethoscope operates in two modes optimized for different frequency ranges:"Heart Mode"(50–300 Hz) and"Lung Mode"(100–1200 Hz). For direct auscultation, audio was recorded in a 16 kHz, mono, 16-bit PCM format. When using the companion application for auscultation or AI analysis, the audio was converted to an 8 kHz, mono, 16-bit PCM format to ensure compatibility with the application’s analytical tools.

### Deep learning algorithms

Audio recordings were collected using the WP-100 digital stethoscope to develop a deep learning-based MR assessment model. In total, 1,840 audio files were obtained from 460 patients, resulting in over 14 h of data from four heart positions per patient. The dataset was then divided into a 7:2:1 ratio, with 321, 92, and 47 patients allocated to the training, validation, and test sets, respectively. This split was based on the conventional practice of splitting datasets into a 7:3 ratio for training and testing, with the test set further divided into validation and test subsets. Stratified random sampling was employed to ensure a balanced class distribution across these sets. The label distributions for mild, moderate, and severe cases were as follows: 18.3%, 45.8%, and 35.9% in the training set; 18.2%, 41.1%, and 40.8% in the validation set; and 13.8%, 48.2%, and 38.0% in the test set, respectively. The hold-out method was used, wherein the validation set monitored the classification performance during training, and the test set was reserved for the final evaluation of the model. The model training and evaluation were performed using the computational resources detailed in [Additional File 1].

The preprocessing pipeline involved segmenting the audio recordings into fixed-length segments of 8 s to ensure uniformity of the input data. Each segment was then transformed into feature representations using either filter bank (Fbank) features or mel spectrograms based on the specified parameters. The transformation parameters, which were consistent for the Fbank and mel spectrogram approaches, included a sample rate of 8,000 Hz, FFT(Fast Fourier transform) size of 1024, 64 mel bands, window length of 1024, and frequency limits of 10–500 Hz. Standard normalization techniques were applied to the audio signals prior to transformation to enhance signal quality and ensure consistency. This normalization, performed automatically by a function in Torchaudio, scales the values within the range of −1.0 to 1.0. Subsequently, the transformation was implemented to capture the essential frequency characteristics of heart sounds while minimizing noise and irrelevant variations.

**Fig. 2:**
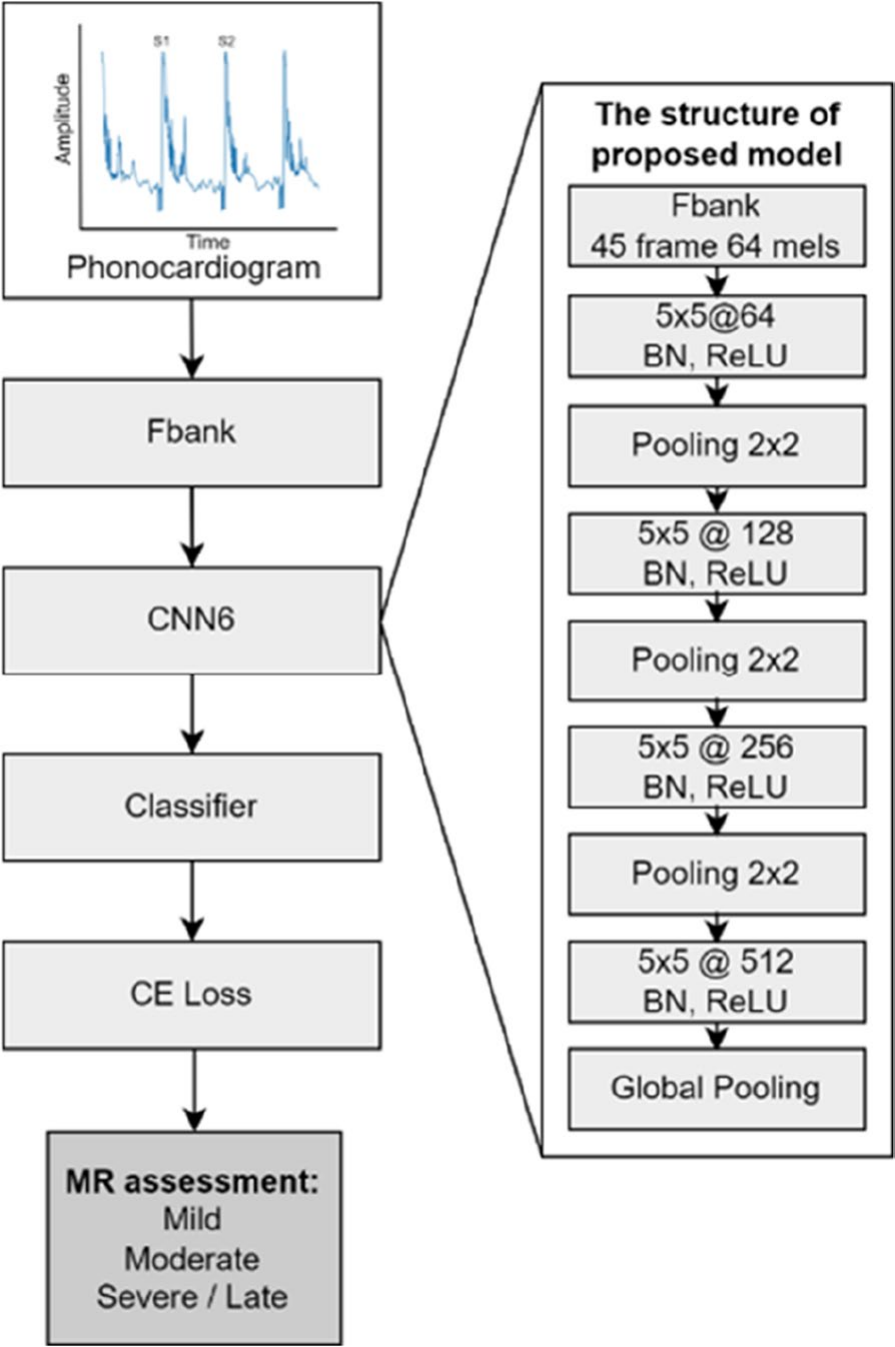
The architecture of the deep learning model for evaluating mitral regurgitation severity. This diagram illustrates how a deep learning model analyzes heart-sound data to categorize the intensity of heart murmurs in dogs. The model uses spectrogram data analyzed by a CNN6 model to determine murmur severity and categorizes it as mild, moderate, or severe. The layer details of the model are presented on the right. CNN, convolutional neural network; Fbank, filter bank; CE, cross-entropy; MR, mitral regurgitation; BN, batch normalization; ReLU, rectified linear unit; FC, fully connected

Three deep learning models were investigated: convolutional neural network (CNN6), residual neural network (ResNet38), and patch-mix audio spectrogram transformer (PaSST). The CNN6 architecture processes the input features through several convolutional layers with 5 × 5 kernels, each followed by batch normalization (BN) and rectified linear unit (ReLU) activation functions. The convolutional layers had increasing filter sizes of 64, 128, 256, and 512, with max-pooling layers of a 2 × 2 kernel size used after each convolutional block to reduce the spatial dimensions. A global pooling layer aggregated the feature maps before passing them to a fully connected classifier layer (Fig. 2, Additional File 2 (A)).

The ResNet38 architecture adhered to the standard ResNet architecture with multiple residual blocks. Each block comprises convolutional layers with 3 × 3 kernels, BN, and ReLU activation. The filter sizes progressively increased, and the network included downsampling and identity connections. Global pooling was applied at the end of the network before a fully connected classifier layer was applied [Additional File 2 (B)].

The PaSST architecture incorporates a transformer-based architecture featuring a linear projector to transform the input features, followed by multiple transformer encoder layers to capture long-range dependencies and temporal patterns in the data. A fully connected classifier layer was used for the final classification [(Additional File 2 (C)].

Each network’s output layer comprised a fully connected layer, followed by three outputs normalized to a probability distribution via a softmax function. The networks were initialized with random weights and optimized using the Adam optimizer. The training was conducted with an initial learning rate of 0.0005 and a batch size of eight over a fixed number of epochs, with early stopping based on validation loss to prevent overfitting. Cross-entropy loss was utilized for all training experiments.

The end-to-end algorithm classified inputs into one of three possible outputs—mild, moderate, or severe—indicating the stage of MR. The final evaluation of the test set provided a comprehensive assessment of the effectiveness of each model for classifying MR severity.

### Performance evaluation

Five classification metrics (accuracy, sensitivity [recall], specificity, precision, and F1 score) were employed to evaluate the performance of the deep learning model for diagnosing the severity of MR in canine patients. Macro-averaging was used for the sensitivity, specificity, precision, and F1 score to ensure that each class was given equal importance, providing a balanced view of the model’s effectiveness across all categories. These metrics provide a thorough evaluation of the model’s performance across different aspects without introducing bias [41].

### Statistical analysis

Data analysis and visualization were performed in Python 3.10 using the standard packages NumPy 1.21.5, Pandas 1.5.2, Seaborn 0.12.2, Matplotlib 3.7.1, Scikit-learn 1.3.0 and Torchaudio 2.0.0. Statistical analyses, including the calculation of standard deviation and computation of 95% confidence intervals (CIs) and receiver operating characteristic (ROC) curves, were performed using the scikit-learn library, Torchmetrics 1.12, and Microsoft Excel 2021.

## Results

### Data analysis process and participant characteristics

This study utilized a systematic approach to collect, preprocess, and analyze the PCG data from dogs with MMVD. Data were acquired using a digital stethoscope, followed by preprocessing to clean and format the data for analysis. The data were then visualized and analyzed using deep learning models, including Fbank, PaSST, ResNet38, and CNN6 (Fig. 1).

**Fig. 3:**
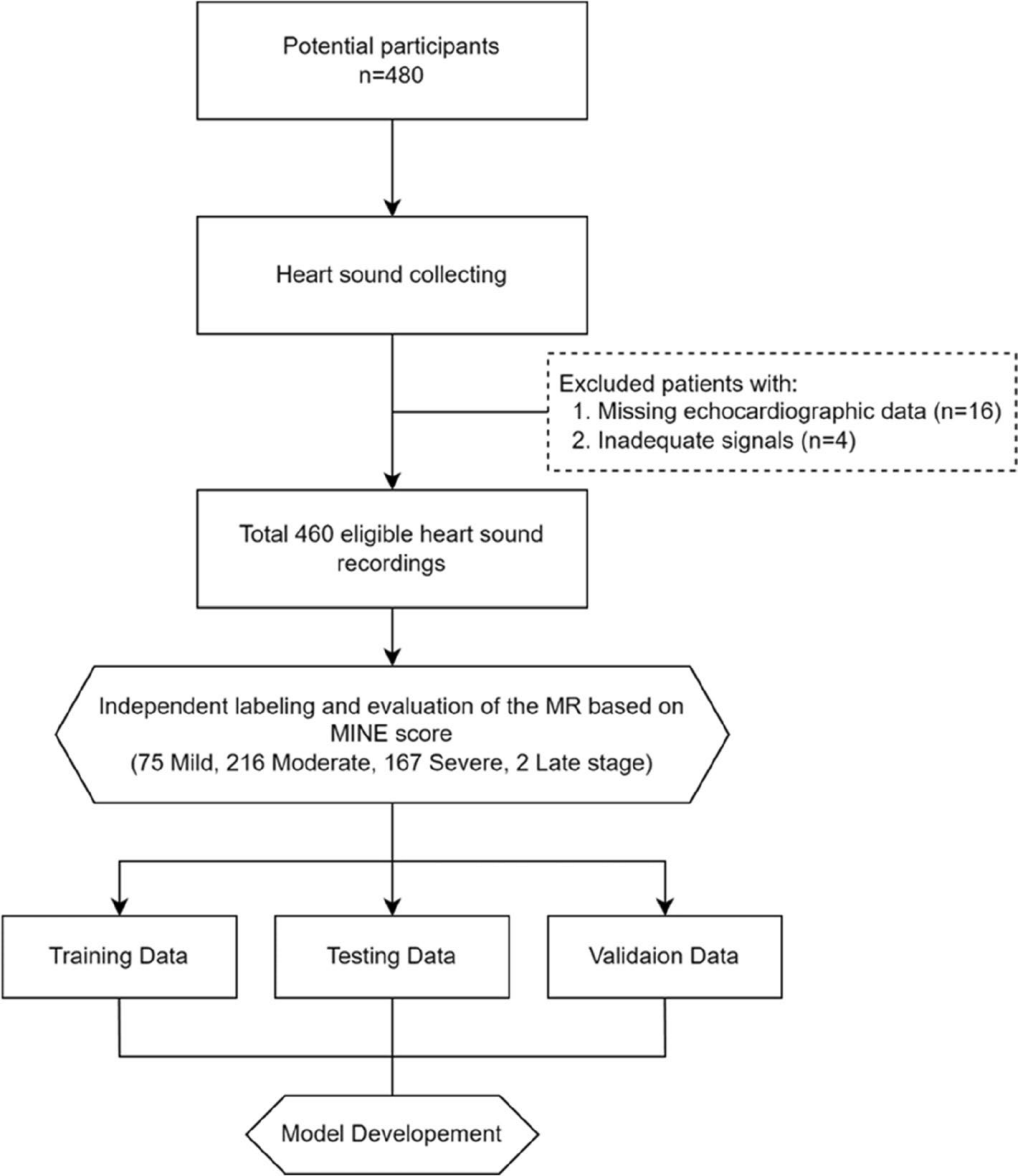
Participant selection and workflow for deep learning model development. The flowchart illustrates the process of collecting and analyzing heart-sound data for the assessment of the severity of mitral regurgitation in participants. Of note, the severe group initially included 167 cases; however, later in the study, two cases previously classified as late-stage were merged into the severe group owing to their small sample size. The recordings were then segmented into training, test, and validation datasets for model development. MINE score, mitral insufficiency echocardiographic score

Participant selection involved 480 potential candidates; 460 were deemed eligible. Exclusions were based on two main criteria: missing echocardiographic data and inadequate signal quality (Fig. 3). Inadequate signal quality was identified through the detection of ambient noise, such as talking, friction sounds from patient movement during measurements, or incomplete recordings where data were not captured in all the necessary positions. Missing echocardiographic data referred to instances wherein the essential values required for the MINE score [2] were unavailable; this could be owing to challenges in performing a full echocardiogram—such as a lack of patient cooperation—specific requests from the owners, or other procedural limitations.

The remaining 460 recordings were labeled using the MINE score and categorized into mild (*n* = 75), moderate (*n* = 216), and severe (*n* = 169) cases. The enrolled dogs were categorized into ACVIM stages as follows: 139 in stage B1, 171 in stage B2, 132 in stage C, and 18 in stage D. These stages were used soley to define the study population and were not utilized for severity classification. The cohort had a mean age of 10.3 ± 3.1 years and a mean body condition score of 5.5 ± 1.2. Demographic and clinical characteristics are presented in Table 1. For model development, the dataset was partitioned into training, validation, and test sets.

**Table 1:** Characteristics of study subjects

Characteristics	All Subjects	Mild	Moderate	Severe
Total subjects	460	75	216	169
Sex				
Male	262	68	100	94
Female	198	7	116	75
Neuter State				
Intact	24	9	6	9
Neutered	436	66	210	160
Breed				
Maltese	126	28	37	61
Pomeranian	96	8	47	41
Poodle	50	10	26	14
Shih-Tzu	50	4	23	23
Chihuahua	35	2	20	13
Cocker Spaniel	3	-	2	1
Pekinese	11	-	11	-
Spitz	8	-	4	4
Schnauzer	8	3	5	-
Dachshund	15	9	4	2
Bichon Frise	4	-	-	4
Coton de Tulear	4	-	4	-
Yorkshire Terrier	3	-	3	-
Mixed	45	10	29	6
Others	2	1	1	-
Age				
mean ± SD, y	12.12 ± 4.92	11.30 ± 2.43	12.45 ± 2.79	12.6 ± 2.28
< 7, y	13	4	5	4
7–9 y	42	8	24	10
9–11 y	76	18	38	20
11–13 y	114	24	46	44
13–15 y	138	13	59	66
> 15, y	70	6	41	23
Body Condition Score				
mean ± SD	5.02 ± 1.30	5.15 ± 1.51	5.07 ± 1.38	4.90 ± 1.07
1–3	48	12	25	11
4–6	341	45	153	143
7–9	64	16	35	13
Echocardiography				
mean ± SD	7.04 ± 1.91	4.48 ± 0.50	6.38 ± 0.48	9.10 ± 1.19
LAAo	1.72 ± 0.36	1.48 ± 0.12	1.55 ± 0.24	2.04 ± 0.32
LVIDDn	1.59 ± 0.63	1.34 ± 0.21	1.48 ± 0.27	1.84 ± 0.24
FS (%)	58.66 ± 26.22	42.01 ± 5.99	58.95 ± 9.73	62.59 ± 9.78
E-peak	0.96 ± 0.29	0.77 ± 0.19	0.85 ± 0.20	1.18 ± 0.28

Data for overall age, body condition score, and echocardiographic measurements are presented as mean ± SD. Numbers (percentages) are presented for specific categories of sex, neuter state, breed, age, and body condition scores. Subjects were categorized as mild, moderate, or severe, with late cases combined owing to the small sample size. Breeds included in the “others” category comprised Miniature pinschers and Italian greyhounds

### Model architecture and performance

The architecture of the deep learning model for analyzing heart-sound data includes several critical components (Fig. 2). The model processes spectrogram data through multiple layers, starting with the input layer. The input audio, sampled in the range of 10–1200 Hz, is segmented into fixed lengths of 8 s, and each segment undergoes preprocessing steps such as normalization performed automatically by a function provided by Torchaudio, which scales values within a range of −1.0 to 1.0 when the audio file is loaded. These preprocessed audio segments are then transformed into feature representations using either filter banks or mel-spectrograms, providing the input features required for deep learning. The convolutional layers extract features from the spectrogram, with each layer followed by a ReLU activation function to introduce nonlinearity into the model. These convolutional layers progressively capture the more complex features of heart-sound data. BN layers are included after the convolutional layers to normalize the output and improve training stability and speed. After extraction, the fully connected layers interpret the features to make final predictions by combining the features learned by the convolutional layers and outputting the classification probabilities. The final fully connected layer outputs the classification results as mild, moderate, or severe.

**Fig. 4:**
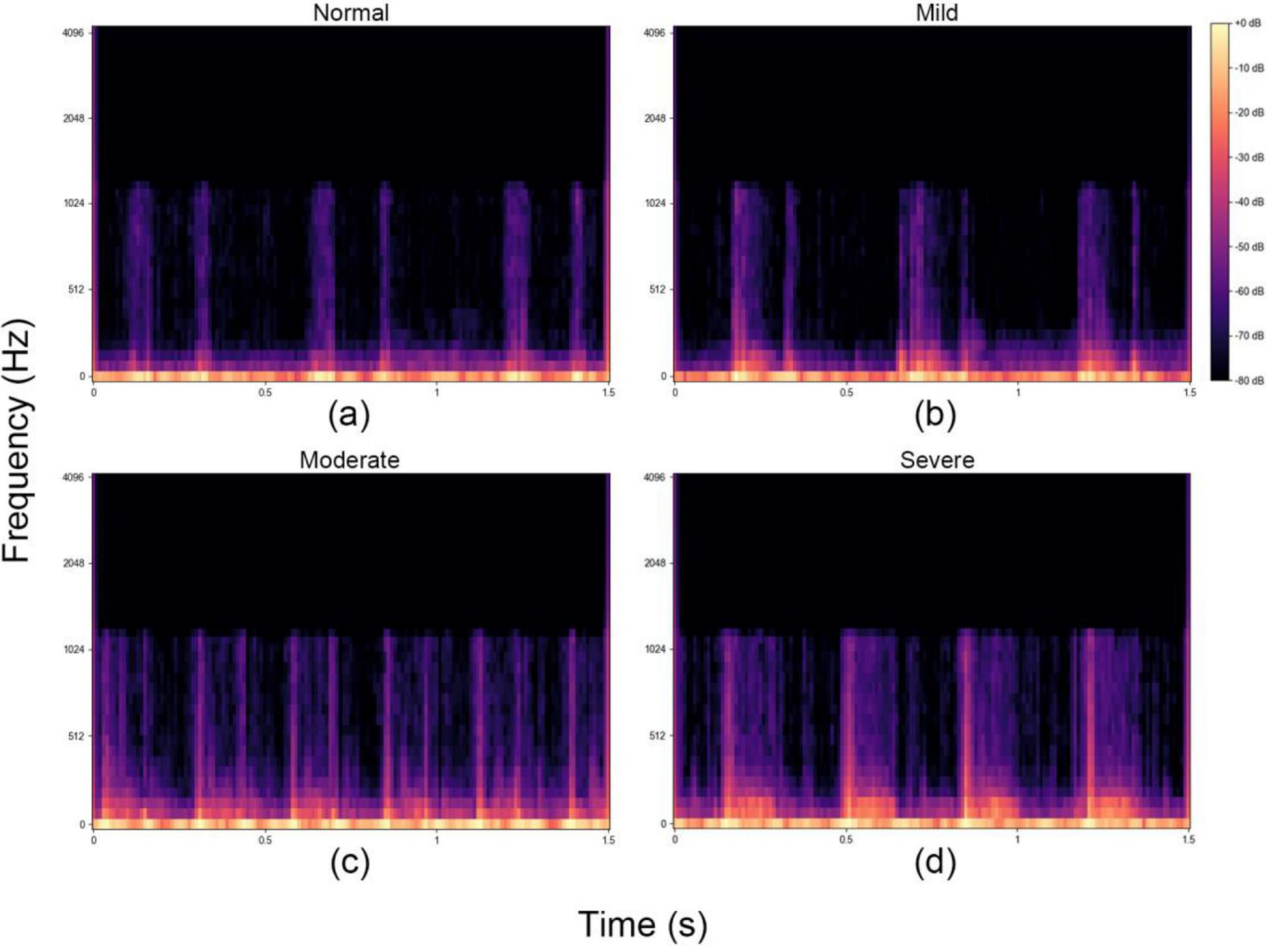
Examples of filter banks. The phonocardiogram trace in the time domain and its power spectral density demonstrate different levels of mitral regurgitation severity, categorized as (A) normal, (B) mild, (C) moderate, and (D) severe

The features used in our deep learning model are automatically extracted by the deep learning architecture during training. The raw PCG signals, after preprocessing and transformation into feature representations, serve as input to the model. PCG traces in the time domain and their corresponding power spectral density plots were examined to determine the different MR severity levels. The deep learning model leverages convolutional layers to identify and extract hierarchical patterns from these representations that are indicative of MR severity. No additional variables or manually engineered features were introduced to the algorithm. Instead, the model learns relevant features directly from the input data during training, optimizing classification performance. This approach aligns with modern practices in deep learning, where feature extraction is seamlessly integrated into the training process rather than relying on predefined statistical or signal-based features. Figure 4 shows examples categorized as normal (Fig. 4A), mild (Fig. 4B), moderate (Fig. 4C), and severe (Fig. 4D). These examples illustrate the variations in PCG signals corresponding to different MR severities, highlighting the distinct patterns used by the model to differentiate between severity levels.

**Fig. 5:**
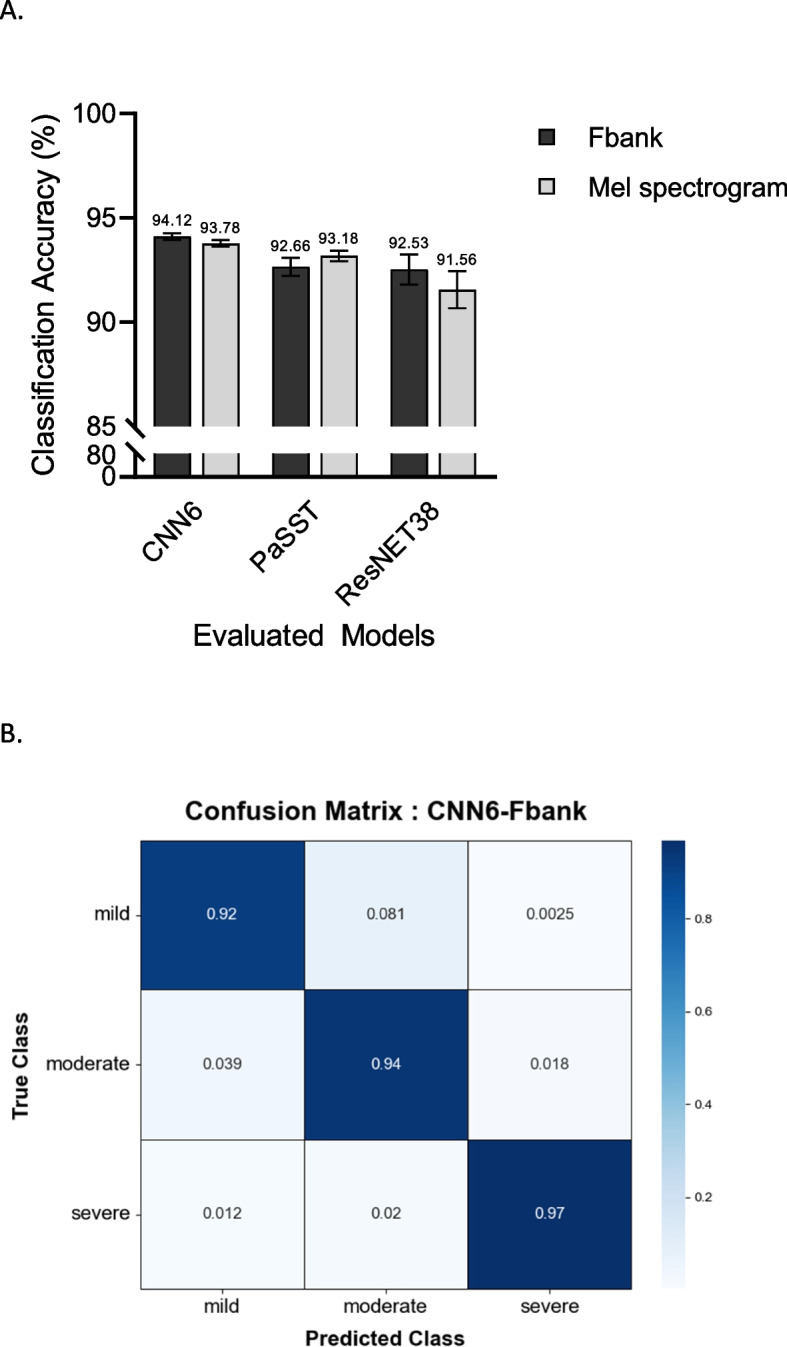
Performance evaluation of deep learning models. A. Classification accuracies achieved by individual models in evaluating mitral regurgitation severity. This graph shows the accuracy of three audio processing models—CNN6, PaSST, and ResNet38—using two types of features to analyze audio data: Fbank and mel spectrograms. Each set of bars illustrates the accuracy results for each model, with lighter shades representing Fbank and darker shades representing mel spectrograms. Standard deviations are indicated by error bars. B. Confusion matrix for CNN-based mitral regurgitation classification. The confusion matrix depicts the performance of our CNN in classifying the severity of mitral regurgitation. The true severity levels are plotted on the y-axis, and the predicted severity levels are plotted on the x-axis. Each cell contains the percentage of instances for each predicted true-label pair. CNN, convolutional neural network; Fbank, filter bank; PaSST, patch-mix audio spectrogram transformer; ResNet, residual neural network

The classification accuracies of the three audio processing models—CNN6, PaSST, and ResNet38—using Fbank and mel spectrogram features showed that CNN6 achieved the highest accuracy with Fbank features, with an average accuracy of 94.12% (95% Cl: 94.11–93.12). These results indicate that regarding accuracy, CNN6 was superior, followed by PaSST and ResNet38 (Fig. 5A). Additional performance metrics for the CNN6, PaSST, and ResNet38 models using the Fbank and mel spectrogram features are summarized in Table 2. The CNN6 model demonstrated the highest overall performance, particularly with Fbank features, achieving a specificity of 97.30% (95% CI: 97.30–97.34), sensitivity of 94.12% (95% CI: 93.74–94.50), precision of 92.63% (95% CI: 92.29–92.97), and F1 score of 93.32% (95% CI: 93.05–93.59). PaSST and ResNet38 had specificities of 96.75% (95% CI: 96.74–96.75) and 96.80% (95% Cl: 96.80–98.81), sensitivities of 90.34% (95% CI: 89.94–90.74) and 88.27% (95% CI: 87.85–88.69), precision scores of 89.45% (95% CI: 88.81–90.09) and 86.78% (95% CI: 86.20–87.36), and F1 scores of 89.89% (95% CI: 89.48–90.30) and 87.52% (95% CI: 87.20–87.84), respectively.

**Table 2:** Performance evaluation of deep learning models

Metric	CNN6		PaSST		ResNet38	
	Fbank	Mel spectrogram	Fbank	Mel spectrogram	Fbank	Mel spectrogram
Accuracy (%)	94.12 (± 0.1431)	93.78 (± 0.1369)	92.66 (± 0.3852)	93.18 (± 0.2279)	92.53 (± 0.6505)	91.56 (± 0.7947)
Specificity (%)	97.3 (± 0.0009)	97.22 (± 0.0011)	96.75 (± 0.0016)	97.02 (± 0.0014)	96.8 (± 0.0016)	96.04 (± 0.0042)
Sensitivity (Recall, %)	94.12 (± 0.1431)	93.78 (± 0.1369)	92.66 (± 0.3852)	93.18 (± 0.2279)	92.53 (± 0.6505)	91.56 (± 0.7947)
Precision (%)	92.63 (± 0.2724)	93.73 (± 0.5896)	92.53 (± 0.5512)	93.43 (± 0.1964)	91.11(± 0.6216)	92.81 (± 0.7480)
F1 score (%)	93.32 (± 0.1806)	93.75 (± 0.3440)	92.59 (± 0.4684)	93.30 (± 0.1880)	91.75 (± 0.5024)	92.14 (± 0.6770)

*LAAo* left atrium-to-aorta ratio, *LVIDDn* left ventricular end-diastolic diameter normalized to body weight, *FS* fractional shortening of the left ventricle, *E-vel* E-wave transmitral peak velocity

This table quantifies the performance of three distinct deep learning models—CNN6, PaSST, and ResNet38—utilizing two audio feature extraction methods: Fbank and mel spectrograms. The performance metrics are reported as the mean ± standard deviation for each model-feature combination. The bold values represent the highest observed metrics across the models for each feature type.

CNN, convolutional neural network; Fbank, filter bank; PaSST, patch-mix audio spectrogram transformer; ResNet38, residual neural network.

The performance of the model was evaluated using a confusion matrix that illustrated the performance of the CNN6-Fbank model in classifying MR severity, demonstrating high accuracy; most instances fell along the diagonal. The deep learning model successfully classified 92% of the mild cases, 94% of the moderate cases, and 97% of the severe cases, indicating its robust capability to accurately identify each severity level. The consistently high performance across all classes demonstrates the effectiveness of the model in distinguishing between different MR severities (Fig. 5B). Additionally, the predictive ability of the model was assessed using the ROC curve, yielding area under the curve values of 0.97, 0.98, and 0.99 for mild, moderate, and severe stages, respectively, further demonstrating the model's exceptional discriminative power (Additional File 3).

**Fig. 6:**
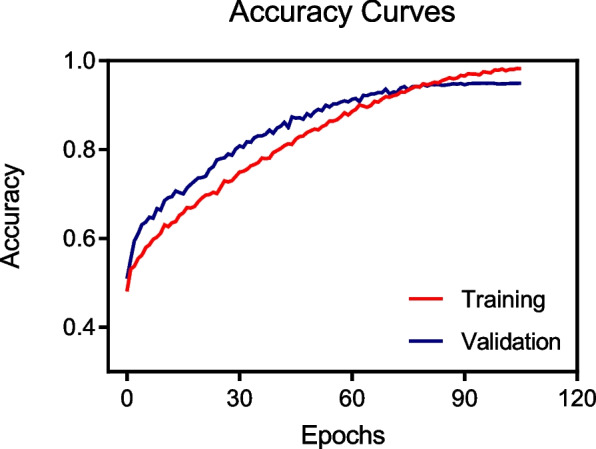
Model training and validation accuracy for evaluating the severity of mitral regurgitation. This figure depicts the accuracy curves during the training and validation phases of the deep learning model designed to evaluate the severity of mitral regurgitation. The blue line represents the training accuracy, whereas the red line represents the validation accuracy. The graph illustrates the progression of accuracy over multiple epochs, highlighting the learning and generalization capabilities of the model

The training and validation accuracy curves of the deep learning model are depicted in Fig. 6, illustrating the model’s performance throughout the training process. The training accuracy exhibited a steady upward trend, ultimately achieving a final accuracy of 0.98, whereas the validation accuracy consistently improved, stabilizing at 0.95 by the final epoch. These findings indicate that the model effectively avoids overfitting and demonstrates strong generalization capabilities to unseen data, ensuring high performance across training and validation datasets.

The results highlight the potential of deep learning models in clinical settings, suggesting that their integration into routine practice could substantially enhance the management and prognosis of dogs with MMVD.

## Discussion

In this study, we introduced a novel approach that integrates deep learning models with digital stethoscopic recordings to evaluate MR severity in dogs with MMVD. Our findings highlight the potential of heart sound recordings—specifically PCG signals—as primary data sources for classification, representing a significant advancement in veterinary cardiology. The relationship between heart murmur and MR [21] and its connection to MMVD severity [42] has been previously explored, offering insights into how acoustic characteristics relate to disease severity. Although murmur intensity is useful for identifying disease stages, it has limitations owing to overlapping categories, particularly in moderate and loud murmurs, which reduce its reliability in tracking disease progression. Building on this context, we applied deep learning techniques to PCG signals, further validating the utility of phonocardiographic data in assessing MR. Although not intended to replace echocardiographic assessments, the proposed system offers a valuable tool for early screening and disease prediction, offering an accessible, cost-effective approach that may provide informative input for clinical decision-making in dogs with MMVD. It should be noted that this prediction model does not incorporate ACVIM staging, which remains the primary framework currently used to guide therapeutic decisions. The CNN6 architecture demonstrated particularly superior accuracy in classifying MR severity levels, outperforming contemporary models such as PaSST and ResNet38. This methodological innovation democratizes diagnostic processes and enhances the feasibility and efficiency of MR severity assessment, potentially broadening access to advanced diagnostic capabilities in diverse clinical settings.

This study further distinguishes itself by focusing on algorithms specifically designed for canine MMVD patients, in contrast to previous research that utilized human-trained recurrent neural networks for heart murmur detection [43]. A notable methodological distinction lies in the representation of phonocardiographic data: although previous study employed log-spectrogram features, our study used Fbank representations. This study builds on previous research in heart sound analysis by applying deep learning models to veterinary diagnostics, demonstrating their adaptability to address various diagnostic challenges in the field.

In human medicine, deep learning models have been successfully applied to the analysis of heart sounds and echocardiographic data, thereby providing enhanced diagnostic capabilities. For instance, research has demonstrated the efficacy of deep learning models in identifying arrhythmias from electrocardiographic (ECG) data, underscoring their potential for improving cardiac diagnostics [44]. The use of digital stethoscopes combined with AI for diagnosing heart conditions has also been well-documented [45]. Studies demonstrating the high accuracy of deep learning models in classifying heart murmurs suggest that these methodologies can also be effectively applied in veterinary medicine [46]. A recent study demonstrated the application of a machine-learning algorithm, originally trained on human data, to canine patients with cardiac disease, effectively grading heart murmurs and distinguishing preclinical stages of MMVD [43]. Despite these promising developments, integration of such advanced technologies into routine veterinary practice remains limited. Our study highlights the potential of deep learning models to reduce the skill level required for accurate auscultation, enabling earlier detection of disease progression, and offering a practical, accessible tool to support clinical decision-making in the management of MMVD.

In this study, we evaluated the effectiveness of the CNN6, ResNet38, and PaSST models in conjunction with Fbank and mel spectrograms for transforming time domain data into frequency domain representations. The experimental findings revealed that when paired with Fbank, the CNN6 model outperformed the other model-feature combinations. CNN6 effectively analyzes heart sounds through the efficient extraction of local features from time-series data; this ability to detect time-series patterns is critical for the precise classification of MR severity [47]. ResNet38 improves classification accuracy by capturing more complex patterns and features with a deeper architecture [48]. By contrast, PaSST employs attention mechanisms that allow the model to focus on critical PCG signals [49].

Our results underscore the superior performance of Fbank over the mel spectrograms. Although mel spectrograms convert frequencies to a logarithmic scale that closely mimics human auditory perception, they may miss subtle variations in certain frequency bands [50]. By contrast, Fbank employs filter bank analysis to extract the frequency components, making it more adept at identifying critical frequency bands [51]. This distinction likely accounts for the superiority of Fbank observed in our experiments.

Furthermore, the study highlights the unique strengths of CNN6 in extracting local features, which proves to be more effective in this context than transformer models—known for their ability to learn global relationships in long-sequence data—but potentially less effective at capturing local patterns. Although ResNet38 structure enables the capture of more complex patterns, it requires more computational resources and carries a higher risk of overfitting.

It can be reasonably deduced that the anatomical and physiological variations inherent to the conformation of the canine chest, as well as the body condition of the animal, have a notable impact on the accuracy of cardiac auscultation and PCG signal. Various factors can influence the intensity of heart sounds, including chest wall thickness, distance between the heart and chest wall, obesity, tachycardia, anemia, and effusion. Specifically, the distance between the heart and chest wall is a significant factor that influences the clarity of heart sounds [52]. Although data augmentation techniques are commonly recommended to improve model performance in this context [53], this study did not employ such methods. The introduction of noise during data augmentation has the potential to compromise deep noise analysis by filtering out critical information, ultimately reducing the model performance [53, 54]. Despite these challenges, data augmentation remains valuable for enhancing medical classification tasks, such as screening and triage. Although this study did not employ data augmentation owing to concerns regarding noise introduction, it has proven to be effective in other areas, such as cardiac sound analysis, by generating synthetic data that accurately reflect physiology. Future technological advancements and the incorporation of clinician input will be crucial for developing models that are accurate and clinically useful [55].

The findings of this study have important implications for the management of canine MMVD. Studies in dogs with MR attributable to MMVD have revealed that increasing MR severity is associated with the characteristic features of PCG recordings. One study identified that changes in heart sounds and murmurs were related to the severity of chronic valvular disease in Cavalier King Charles Spaniels [56]. Although some studies have reported variability in the auscultation of mild MR in dogs, considering factors such as the effects of physical maneuvers and agreement with color Doppler echocardiography and PCG^19^, it is generally accepted that as the severity of MR increases, the murmur tends to extend from early or late systole to holosystolic [56], with a corresponding increase in the amplitude and frequency of the murmur. The CNN6 model leverages these distinctions by identifying specific features within heart sounds that correlate with MR severity, such as the intensity and frequency of murmurs [57], providing a non-invasive and efficient method for assessing MR. As MMVD progresses, the severity of regurgitation increases, making regular monitoring vital for effective disease management.

The high classification accuracy of the CNN6 model demonstrates the potential of deep learning-assisted auscultation tools to provide reliable and rapid assessments of MR severity [58]. This may provide prognostic insight, aiding veterinarians in assessing prognosis and making more informed clinical decisions, thereby potentially contributing to improved patient outcomes. These findings suggest that the use of PCG signals for MR assessment could potentially complement echocardiographic evaluations by simplifying the diagnostic process and improving accessibility. Nevertheless, further investigation is required to evaluate how effectively this deep learning model can detect changes in MR severity and facilitate timely veterinary intervention, ensuring its practical utility in clinical and diagnostic settings.

Accurate assessment and monitoring are critical for managing cardiac diseases, and digital stethoscopes with AI-assisted auscultation provide a practical solution by detecting subtle changes in heart health and supporting the management of conditions like MMVD^23^. However, the adoption of such technologies is often limited by high costs and the need for specialized training. Affordable alternatives, such as deep learning-enhanced digital stethoscopes, address these challenges by combining accessibility with diagnostic reliability, bridging the gap between innovation and practicality to improve outcomes in routine clinical practice [56, 59, 60].

This study also investigated the explainability of the CNN6 model's predictions using gradient-weighted class activation mapping (Grad-CAM). Grad-CAM visualizations identified areas of high activation in spectrogram regions corresponding to transformed PCG signals, indicating that the model predominantly relied on heart sounds for its predictions [Additional File 4]. However, the approach was limited in its ability to pinpoint specific PCG characteristics—such as murmur intensity, the S1/S2 amplitude ratio, and other features previously associated with MR severity—that informed the model's decisions [21]. This limitation arises from the Fbank transformation, which applies overlapping windows and fixed Mel bins, reducing temporal resolution and hindering the direct attribution of specific temporal features to the model's predictions.

These challenges highlight the inherent"black box"nature of deep learning models, where the underlying decision-making processes are often opaque and difficult to interpret. Such opacity raises critical concerns regarding clinical accountability, particularly in diagnostic applications where understanding the basis of a model’s decisions is essential to ensure safety and reliability [61, 62].

Expanding on these results, our next steps will focus on integrating explainable AI methodologies capable of uncovering the specific PCG features that drive model predictions. These advancements will enhance transparency and provide deeper insights into how deep learning models utilize PCG data, ultimately refining their diagnostic utility and fostering greater trust in their application for managing canine MMVD.

Despite the promising results, some limitations should be considered. First, the data were collected exclusively from a single veterinary clinic, which may limit the generalizability of the findings to other clinical settings. Second, the relatively small sample size and insufficient representation of late-stage MMVD cases constrained the model’s ability to fully address advanced disease scenarios, underscoring the need for validation in larger and more diverse populations. Furthermore, the study focused solely on MMVD patients, excluding healthy controls, which may have limited the model's ability to generalize across a broader spectrum of clinical conditions. Another important consideration is that the heart sound recordings were obtained under controlled clinical conditions by specialized veterinary practitioners. Variability in recording environments, practitioner expertise, and device settings was not accounted for, which may affect the model’s robustness in real-world scenarios. In addition, pharmacological treatment was ongoing in a subset of dogs at the time of data collection, potentially affecting echocardiographic parameters such as LA/Ao and LVIDDn. These treatment-related factors may have contributed to variability in the echocardiographic findings, complicating the interpretation of MINE scores.

Finally, the analysis was based on the MINE classification system, which offers valuable prognostic insights, but is not aligned with other widely adopted frameworks such as the ACVIM consensus statement. The ACVIM consensus statement offers definitive guidance for differentiating between stage B1 and B2, a pivotal decision point for initiating treatment. In contrast, the MINE classification, based on echocardiographic parameters, does not address this critical clinical threshold, limiting its applicability for early intervention. Additionally, reliance on the MINE classification excludes diagnostic tools such as thoracic radiography and clinical assessments, which are essential for identifying stage C and D. These limitations constrain the scope of the findings and reduce their utility in veterinary practice.

Addressing these limitations in future studies will be critical to improving the robustness and applicability of the proposed algorithm. Among these, pharmacological factors warrant particular attention. Although treatment variables were not included in the current analysis, future models may benefit from integrating pharmacological data. In particular, the use of diuretics, which directly influences heart size, could be incorporated as a relevant clinical parameter to enhance the clinical reliability of the algorithm. Efforts should also include multisite data collection across diverse clinical environments, larger and more heterogeneous patient populations, and the inclusion of MMVD patients and healthy controls. Expanding the dataset and incorporating multimodal diagnostic parameters, such as echocardiographic, electrocardiographic, and radiographic data, could further enhance the model’s accuracy and clinical utility. Evaluating the model in real-world clinical workflows will also provide valuable insights into its practical implementation and impact on decision-making.

## Conclusion

Our study demonstrated that deep learning models, particularly CNN6, can potentially assess MR severity in canine MMVD using digital stethoscope recordings. This methodology, which involves the analysis of heart sounds, offers a rapid and straightforward supplementary approach to echocardiography, thereby enhancing its diagnostic efficacy. Despite some limitations, these findings highlight the possibility that incorporating deep learning could enhance veterinary cardiology, with further validation and real-time applications potentially improving diagnostic precision and patient outcomes.

## Supplementary Information

Additional file 1: Hardware and software environment details. This table summarizes specifications of the computational setup used for data analysis, training and evaluation

Additional file 2: The architecture of the deep learning models for evaluating mitral regurgitation severity using other methods.This figure illustrates the architectures of three supplementary models used for mitral regurgitation (MR) assessment that are not represented in the main data: (A) CNN6-Mel spectrogram model, (B) PaSST-based model, and (C) ResNet38-based model. In each model, phonocardiogram (PCG) signals are first transformed into filter bank (Fbank) or Mel spectrogram representations. These representations are then processed through the respective models—PaSST, ResNet38, and CNN6—which consist of various layers and structures to determine murmur severity and categorize it as mild, moderate, or severe. The model layer details are shown on the right

Additional file 3: Receiver operating characteristic curves for mitral regurgitation severity classification using the CNN6-Fbank model.This figure presents the receiver operating characteristic (ROC) curves for the CNN6-Fbank model used to evaluate the severity of mitral regurgitation (MR). The ROC curves depict the performance of the model in classifying MR severity into three categories: mild, moderate, and severe. The area under the curve (AUC) values for each category are as follows: mild (AUC=0.97), moderate (AUC=0.98), and severe (AUC=0.99)

Additional file 4: Grad-CAM visualization of the CNN6-Fbank model for mitral regurgitation classification. This figure depicts the regions of interest identified by deep learning models for classifying the severity of mitral regurgitation. The color intensity represents the relative contribution of each region to the model’s decision, with warmer colors (e.g., red) indicating higher importance and cooler colors (e.g., blue) indicating lower importance. (a) Mild, (b) Moderate, and (c) Severe

## Data Availability

Data is provided within the manuscript or supplementary information files.

## Abbreviations

ACVIM: American College of Veterinary Internal Medicine
AI: Artificial Intelligence
BN: Batch Normalization
CHF: Congestive Heart Failure
CNN: Convolutional Neural Network
ECG: Electrocardiographic
Fbank: Filter Bank
MINE: Mitral Insufficiency Echocardiographic Score
MMVD: Myxomatous Mitral Valve Disease
MR: Mitral Regurgitation
PaSST: Patch-mix Audio Spectrogram Transformer
PCG: Phonocardiography
ReLU: Rectified Linear Unit
ResNet: Residual Neural Network
ROC: Receiver Operating Characteristic
SNU VMTH: Seoul National University Veterinary Medicine Teaching Hospital
