# Supplementary material for: Deep learning-based evaluation of the severity of mitral regurgitation in canine myxomatous mitral valve disease patients using digital stethoscope recordings
Source: BMC Vet Res. 2025 May 8;21:326. doi: 10.1186/s12917-025-04802-z (PMC12060408; doi:10.1186/s12917-025-04802-z)
Supplement: Supplementary file 1 — Additional file 1: Hardware and software environment details. This table summarizes specifications of the computational setup used for data analysis, training and evaluation [file 12917_2025_4802_MOESM1_ESM.docx]

**Additional Files**

**Table 1**. Hardware and software environment details.

| Specification | |
| --- | --- |
| OS | Ubuntu 22.04.2 LTS |
| CPU | 13th Gen Intel(R) Core(TM) i9-13900KF |
| RAM | 62GB |
| GPU | 2 × NVIDIA GeForce RTX 4090 |
| CUDA Version | CUDA 11.8 |

This table summarizes specifications of the computational setup used for data analysis, training and evaluation.
