# Supplementary material for: Deep learning-based evaluation of the severity of mitral regurgitation in canine myxomatous mitral valve disease patients using digital stethoscope recordings
Source: BMC Vet Res. 2025 May 8;21:326. doi: 10.1186/s12917-025-04802-z (PMC12060408; doi:10.1186/s12917-025-04802-z)
Supplement: Supplementary file 2 — Additional file 2: The architecture of the deep learning models for evaluating mitral regurgitation severity using other methods.This figure illustrates the architectures of three supplementary models used for mitral regurgitation (MR) assessment that are not represented in the main data: (A) CNN6-Mel spectrogram model, (B) PaSST-based model, and (C) ResNet38-based model. In each model, phonocardiogram (PCG) signals are first transformed into filter bank (Fbank) or Mel spectrogram representations. These representations are then processed through the respective models—PaSST, ResNet38, and CNN6—which consist of various layers and structures to determine murmur severity and categorize it as mild, moderate, or severe. The model layer details are shown on the right [file 12917_2025_4802_MOESM2_ESM.docx]

**Additional Files**

A.


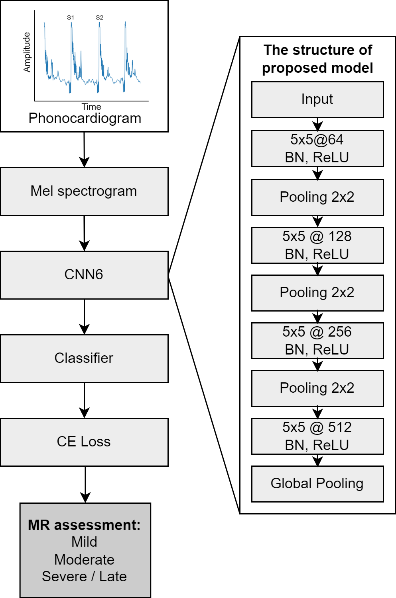


B.


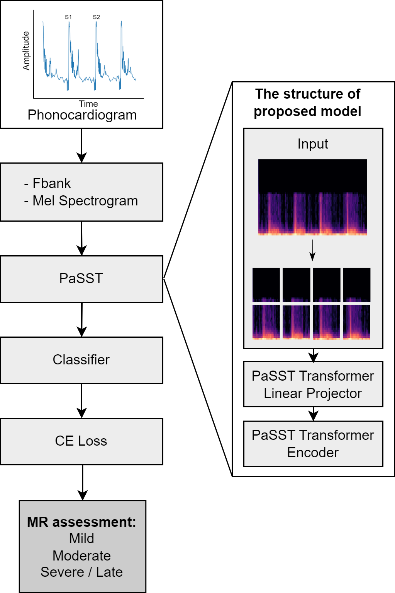


C.


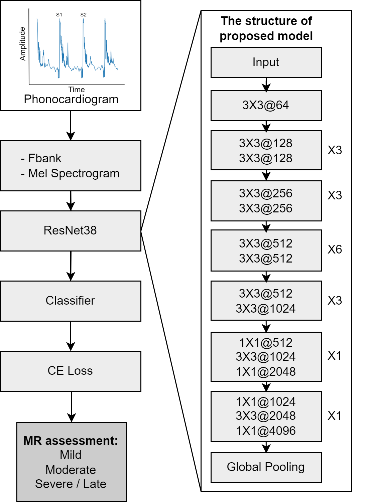


**Additional Figure 1.** **The architecture of the deep learning models for evaluating mitral regurgitation severity using other methods.**

This figure illustrates the architectures of three supplementary models used for mitral regurgitation (MR) assessment that are not represented in the main data: (A) CNN6-Mel spectrogram model, (B) PaSST-based model, and (C) ResNet38-based model. In each model, phonocardiogram (PCG) signals are first transformed into filter bank (Fbank) or Mel spectrogram representations. These representations are then processed through the respective models—PaSST, ResNet38, and CNN6—which consist of various layers and structures to determine murmur severity and categorize it as mild, moderate, or severe. The model layer details are shown on the right.
