# Supplementary material for: Deep learning-based evaluation of the severity of mitral regurgitation in canine myxomatous mitral valve disease patients using digital stethoscope recordings
Source: BMC Vet Res. 2025 May 8;21:326. doi: 10.1186/s12917-025-04802-z (PMC12060408; doi:10.1186/s12917-025-04802-z)
Supplement: Supplementary file 3 — Additional file 3: Receiver operating characteristic curves for mitral regurgitation severity classification using the CNN6-Fbank model.This figure presents the receiver operating characteristic (ROC) curves for the CNN6-Fbank model used to evaluate the severity of mitral regurgitation (MR). The ROC curves depict the performance of the model in classifying MR severity into three categories: mild, moderate, and severe. The area under the curve (AUC) values for each category are as follows: mild (AUC=0.97), moderate (AUC=0.98), and severe (AUC=0.99) [file 12917_2025_4802_MOESM3_ESM.docx]

**Additional Files**

**
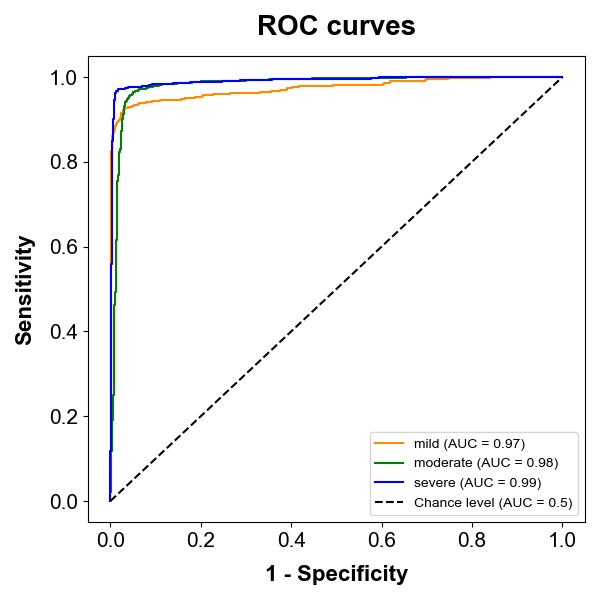
**

**Additional Figure 2.** **Receiver operating characteristic** **curves for mitral regurgitation severity classification using the CNN6-Fbank model**. This figure presents the receiver operating characteristic (ROC) curves for the CNN6-Fbank model used to evaluate the severity of mitral regurgitation (MR). The ROC curves depict the performance of the model in classifying MR severity into three categories: mild, moderate, and severe. The area under the curve (AUC) values for each category are as follows: mild (AUC=0.97), moderate (AUC=0.98), and severe (AUC=0.99).
