# Supplementary material for: Deep learning-based evaluation of the severity of mitral regurgitation in canine myxomatous mitral valve disease patients using digital stethoscope recordings
Source: BMC Vet Res. 2025 May 8;21:326. doi: 10.1186/s12917-025-04802-z (PMC12060408; doi:10.1186/s12917-025-04802-z)
Supplement: Supplementary file 4 — Additional file 4: Grad-CAM visualization of the CNN6-Fbank model for mitral regurgitation classification. This figure depicts the regions of interest identified by deep learning models for classifying the severity of mitral regurgitation. The color intensity represents the relative contribution of each region to the model’s decision, with warmer colors (e.g., red) indicating higher importance and cooler colors (e.g., blue) indicating lower importance. (a) Mild, (b) Moderate, and (c) Severe [file 12917_2025_4802_MOESM4_ESM.docx]

**Additional Files**


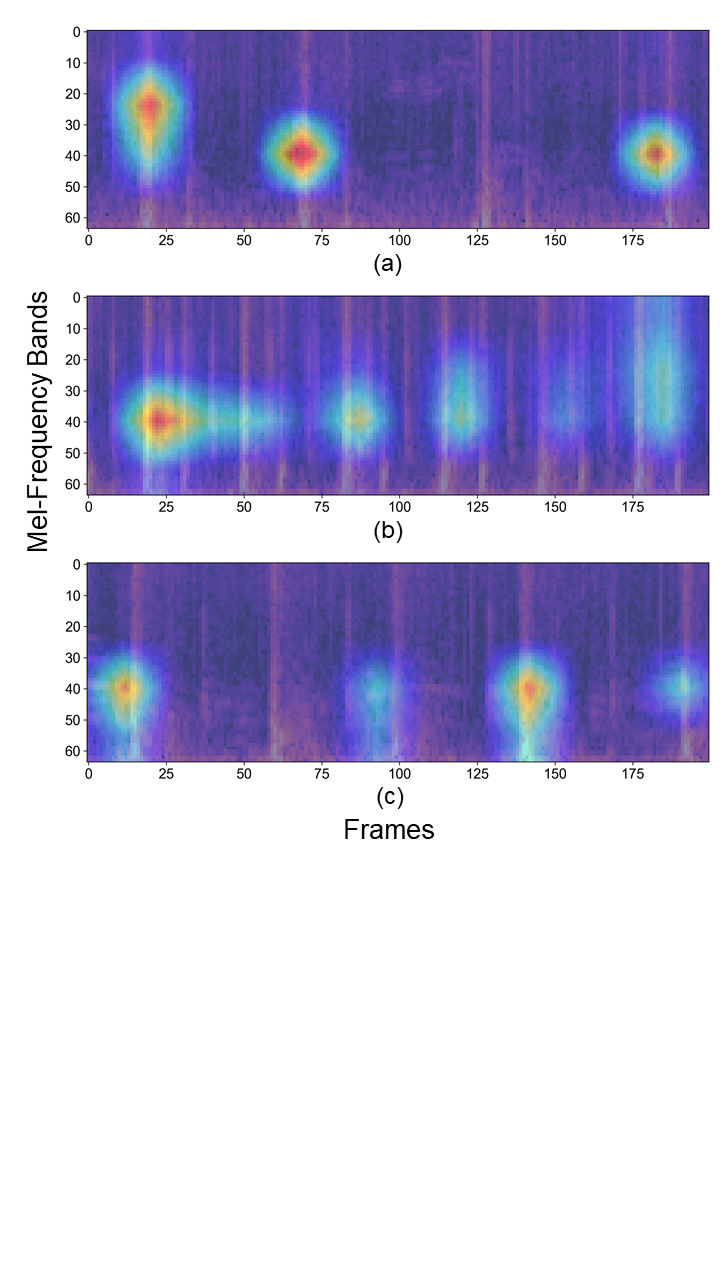


**Additional Figure 3**. **Grad-CAM visualization of the CNN6-Fbank model for mitral regurgitation classification.**

This figure depicts the regions of interest identified by deep learning models for classifying the severity of mitral regurgitation. The color intensity represents the relative contribution of each region to the model’s decision, with warmer colors (e.g., red) indicating higher importance and cooler colors (e.g., blue) indicating lower importance. (a) Mild, (b) Moderate, and (c) Severe.
